# As(V) and As(III) sequestration by starch functionalized magnetite nanoparticles: influence of the synthesis route onto the trapping efficiency

**DOI:** 10.1080/14686996.2020.1782714

**Published:** 2020-07-29

**Authors:** Mbolantenaina Rakotomalala Robinson, Romain Coustel, Mustapha Abdelmoula, Martine Mallet

**Affiliations:** CNRS, LCPME, Université de Lorraine, F-54000 Nancy, France

**Keywords:** Magnetite, polysaccharide, arsenic, sorption capacity, XPS, zeta potential, 212 Surface and interfaces, 301 Chemical syntheses / processing, 308 Materials resources / recycling, 501 Chemical analyses, 502 Electron spectroscopy

## Abstract

We report the effect of the synthesis route of starch-functionalized magnetite nanoparticles (NPs) on their adsorption properties of As(V) and As(III) from aqueous solutions. NP synthesis was achieved by two different routes implying the alkaline precipitation of either a mixed Fe^2+^/Fe^3+^ salt solution (MC samples) or a Fe^2+^ salt solution in oxidative conditions (MOP samples). Syntheses were carried out with starch to Fe mass ratio (R) ranging from 0 to 10. The crystallites of starch-free MC NPs (14 nm) are smaller than the corresponding MOP (67 nm), which leads to higher As(V) sorption capacity of 0.3 mmol g_Fe_^−1^ to compare with respect to 0.1 mmol g_Fe_^−1^ for MOP at pH = 6. MC and MOP starch-functionalized NPs exhibit higher sorption capacities than a pristine one and the difference in sorption capacities between MOP and MC samples decreases with increasing R values. Functionalization tends to reduce the size of the magnetite crystallites and to prevent their agglomeration. Size reduction is more pronounced for MOP samples (67 nm (R0) to 12 nm (R10)) than for MC samples (14 nm (R0) to 9 nm (R10)). Therefore, due to close crystallite size, both MC and MOP samples, when prepared at R = 10, display similar As(V) (respectively, As(III)) sorption capacities close to 1.3 mmol g_Fe_^−1^ (respectively, 1.0 mmol g_Fe_^−1^). Additionally, according to the effect of pH on arsenic trapping, the electrostatic interactions appear as a major factor controlling As(V) adsorption while surface complexation may control As(III) adsorption.

## GRAPHICAL ABSTRACT

**Figure:**
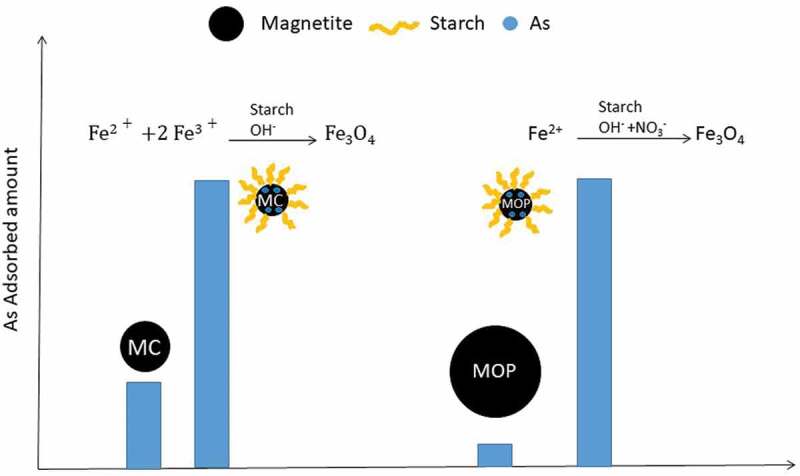

## 1. Introduction

Arsenic is the 20th most abundant element in the earth’s crust and is naturally encountered in igneous and sedimentary rocks. Arsenic is introduced to natural waters from both rocks weathering and anthropogenic sources and mostly from mining and industrial disposal waste, thus posing a real risk to human health. Arsenic is recognized by the World Health Organization (WHO) and some other international organizations among the top 20 priority pollutants. The WHO guideline value and the European Maximum Permissible Concentration (MPC) in water are fixed at 10 µg L^−1^. However, the threshold limit still remains at 50 µg L^−1^ in many countries such as Bangladesh, India, Pakistan, China, and Taiwan making arsenic contamination in drinking water a major problem.

In soils and water, As is predominantly encountered as oxyanions in two oxidation states: arsenite (As(III)) and arsenate (As(V)) with specific forms being dependent on pH and redox conditions. It should be noted that both arsenic forms are toxic but arsenite has been reported 25–60 times more toxic and mobile than arsenate [1,2]. In anyway, simultaneous removal of As(III) and As(V) is usually required in water drinking treatment.

A range of arsenic removal methods are available such as ion exchange, membrane technologies, oxidation, and coagulation-flocculation [1,3–5]. However, these methods cannot be easily implemented in developing countries in view of their high cost and technological complexity. Arsenic removal by adsorption processes has thus gained considerable interest these last decades and appears as the most promising technique due to its high efficiency, easy operation and cost effectiveness. Arsenic adsorption has been studied using a wide range of adsorbents, including silica, activated alumina, activated carbon, and iron oxides [6–8]. In particular, the importance of iron oxides in controlling the mobility and concentration of arsenic species in aqueous media is now well established in the literature [9,10]. More recently, there has been growing interest in studying nanoscale iron oxides in the treatment of arsenic-contaminated water and environmental remediation as nanoparticles combine ‘large’ specific surface area, high reactivity and specificity [11,12]. However, one important limitation is that pure nanoparticles tend to aggregate in larger particles leading to the decrease of specific surface area and reactivity. One solution is therefore to stabilize the iron nanoparticles by grafting or coating with organic molecules including surfactants, polymers and biomolecules. Many literature reports show that well stabilized *i.e*. dispersed iron oxide nanoparticles offer a greater specific surface area and sorption capacity than nanoparticles used without any stabilizer towards a wide range of pollutants [13–17]. This strategy not only stabilizes the magnetite nanoparticles and prevents them from oxidation but may also provide enhanced surface reactivity by adequate functionalization.

Here we propose to evaluate starch-functionalized magnetite (Fe_3_O_4_) nanoparticles for As(V) and As(III) removal. Magnetite is a ubiquitous iron oxide that occurs in the lithosphere, pedosphere and biosphere and contains both ferrous and ferric iron species. Among the available magnetite functionalizing agents, starch, a polysaccharide, appears very attractive owing to the fact that it is among the most abundant and renewable biopolymers, it is a low cost and an environmentally friendly material. Starch-stabilized magnetite nanoparticles were prepared by adapting two standard synthesis protocols and were then compared for As(V) and As(III) removal. The first protocol consisted in the coprecipitation of Fe(II) and Fe(III) ions under alkaline conditions and in the presence of starch while the second route implied alkaline precipitation of Fe(II) in oxidative conditions [15,16]. Previous studies devoted to arsenic remediation only used the co-precipitation approach to synthesize magnetite nanoparticles [10,11]. The primary objective of the study was therefore to understand how the synthesis procedure affects the reactivity and the sorption capacities of starch-functionalized magnetite nanoparticles. It must be mentioned that to the best of our knowledge, this is the first attempt to investigate the influence of the starch-functionalized magnetite synthesis procedure, *i.e*. coprecipitation vs oxidation approach, on arsenic removal efficiency. The starch to iron ratio was a key parameter that was deeply evaluated. More specifically, the effects of pH, adsorbent dose, and initial As(III) and As(V) concentrations were determined. The effects of phosphate and sulfate as competing anions and the arsenic adsorption kinetics were also determined.

## 2. Materials and methods

All chemicals were analytical grade and all solutions were prepared in ultra-pure water (18.2 MΩ cm^−1^) that was bubbled at least for 1 h with N_2_ prior to use. Reaction vessels were washed with dilute HCl (~1%) and rinsed several times with ultra-pure water before use. The As(V) and As(III) stock solutions were prepared from sodium arsenate (NaH_2_AsO_4_) and sodium arsenite (NaAsO_2_) respectively.

### 2.1. Synthesis of starch-stabilized magnetite nanoparticles

*Route 1-Co-precipitation of Fe(II) and Fe(III*): 581 mg of FeSO_4_ · 7H_2_O (2.1 mmol) and 1130 mg of FeCl_3_ · 6H_2_O (4.2 mmol) were dissolved in 70 mL of a 0.1 M HCl solution under magnetic stirring and N_2_ atmosphere (Fe^3+^:Fe^2+^ molar ratio of 2:1). A starch amount was then mixed to the Fe^3+^/Fe^2+^ solution to achieve starch to Fe mass ratio (R) varying over the range between 0.31 (m_starch_ = 109 mg) and 10 (m_starch_ = 3500 mg). The mixture was then heated at 90°C until the gelification of starch was completed. 10 mL of an NH_3_ solution was then rapidly added. The mixture was stirred and heated for an additional 10 minutes. The reaction vessel containing the black precipitate was then cooled overnight. Then, the pH was lowered to 7 with HCl.

*Route 2: Fe(II) precipitation in oxidative conditions*: 80 g of FeSO_4_ · 7H_2_O and the required amount of starch (R ratio in the range 0.1 to 10) were dissolved in 560 mL ultra-pure water under magnetic stirring and N_2_ atmosphere in a 1 L flask and heated up to 90°C. When the temperature was reached, 240 mL of aqueous solution containing 6.46 g KNO_3_ and 44.90 g KOH was added dropwise to the reaction vessel (50 mL min^−1^) using a peristaltic pump. After the addition of the last drop, the mixture was kept heated for 45 minutes.

Finally, the black precipitates obtained in routes 1 and 2 were recovered by centrifugation, washed 2 times with water and finally 1 time with ethanol (96%, from VWR) to remove the unreacted reagents. The resulting product was dried under vacuum.

The obtained composite materials from route 1 were labeled MC_R0 to _R10 while those obtained from route 2 were labeled MOP_R0 to _R10.

### 2.2. Arsenic sorption experiments

Batch arsenic adsorption experiments were carried out in 0.1 M KCl electrolyte and at room temperature. Unless otherwise specified, the adsorbent dose was set at 1.5 g L^−1^, the initial arsenic concentration was 0.1 mM and experiments were carried out at pH 6. Except in kinetic studies, the agitation time was fixed at 24 hours. This time was determined on the basis of a preliminary experiment to be sufficient to reach equilibrium conditions. Requisite quantity of adsorbent was added to 25 mL of As(V) or As(III) solution prepared at the desired concentration.

More specifically, the effect of adsorbent dose on As(V) trapping was determined in the range of 0.5 to 1.5 g L^−1^ and at pH 6. Effects of pH on both As(V) and As(III) adsorption was studied in the range of pH 2–10 by adjusting the pH with NaOH or HCl. Isotherms and kinetics studies were carried out by varying the initial As(V) and As(III) concentrations from 0.02 to 0.54 mmol L^−1^ and contact time from 0 to 1500 min, respectively. The effect of usual competing anions on As(V) and As(III) removal was carried out using 0.1 mM of sulfate or phosphate anions.

At the end of the experiments, samples were filtered using 0.2 µm syringe filters and the residual concentration of arsenic was analyzed using an inductively coupled plasma atomic emission spectrometer.

The adsorption capacity (q_e_) was determined from the following equation:

(1)
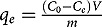

where q_e_ is the amount of adsorbed arsenic (mg g_Fe_^−1^), C_o_ and C_e_ are the initial and equilibrium concentrations of arsenic in solution (mg L^−1^), V is the volume of solution (L) and m is the mass of iron in the adsorbent (g).

### 2.3. Characterization of the starch-functionalized magnetite nanoparticles

The surface physicochemical properties of starch-functionalized magnetite were characterized by X-ray photoelectron spectroscopy (XPS). Spectra were recorded on a KRATOS Axis Ultra X-ray photoelectron spectrometer equipped with a monochromatic Al Kα source (1486.6 eV) operated at power level of 120 W. Powder samples were deposited onto a copper adhesive tape. The analysis area was equal to 300 × 700 µm^2^. The spectra were collected at a take-off angle of 90° and the pressure in the analysis chamber was about 10^−9^ mbar. A pass energy of 20 eV was used for the high-resolution spectra. Charge compensation was applied by flooding low-energy electrons. Binding energies were calibrated by assigning the adventitious carbon C 1 s peak to 284.6 eV. Curve fitting was performed using a Gaussian/Lorentzian (70/30) peak shape after Shirley’s background subtraction and using X–vision 2.2.11 software.

Zeta (ξ) potential of the starch-functionalized magnetite nanoparticles was determined using the Zetasizer Ultra-ZS (Malvern instruments Corp., Malvern, Worcestershire, UK). Measurements were performed on ~1 mL of 0.1 g L^−1^ starch-functionalized nanoparticle suspensions prepared at the desired pH from 2 to 11 by adding small amounts of NaOH or HCl 0.1 M. Suspensions were sonicated ~2 min before measurements.

^57^Fe Mössbauer spectra were collected using a conventional spectrometer in transmission geometry coupled with a cold head cryostat from Advances Research Systems (USA), equipped with vibration isolation stand, developed at the LCPME laboratory. A radioactive source comprising 50 mCi ^57^Co in Rh matrix was mounted in a constant acceleration velocity transducer. Measurements were taken at velocities ranging from −11 to +11 mm s^−1^. The hyperfine interaction parameters were determined by fitting the experimental using the software Recoil and its Voigt-based fitting model [18]. The center shifts are given with respect to metallic α-iron foil at room temperature.

## 3. Results and discussion

### 3.1. Characterization of starch-functionalized magnetite adsorbents

The structural properties of starch-functionalized and pristine magnetite nanoparticles obtained from routes 1 and 2 were characterized in details elsewhere [19]. The cubic spinel structure of magnetite was confirmed by X-ray diffraction (XRD; data not shown). The crystallite sizes, estimated by the Scherrer equation, are summarized in Table 1 as a function of the synthesis route and R ratio. Pristine magnetite nanoparticles prepared from route 1 (MC_R0) exhibits a smaller crystallite size than those obtained from route 2 (MOP_R0) by almost a factor of 5. Salviano et al. [20] have accordingly reported an increase in crystallite size by using oxidation of a Fe(II) solution instead of a mixture of Fe(II) and Fe(III) for the preparation of pristine magnetite particles. In addition, a decrease in crystallite size is clearly induced by starch and this is much more pronounced for magnetite particles prepared by oxidation of Fe(II) (route 2). In fact, samples obtained from route 1 (MC_R0 to R10) present crystallite sizes ranging from 14 to 9 nm while those obtained from route 2 (MOP_R0 to R10) display crystallite sizes ranging from 64 to 12 nm with increasing R ratios. A detailed XRD and transmission electron microscopy (TEM) characterization of MC and MOP NPs has been reported previously [19,21]. In particular, the obtained results by using MET are in agreement with XRD showing a decrease in the nanoparticles size from 81 ± 30 nm for M0P_R0 to about 10 nm for M0P_R10 samples and 13 ± 3 for both MC _R0 and _R10, respectively.

**Table 1.** Crystallite size as a function of R = m_starch_/m_Fe_ ratio for MOP and MC samples.

Route	Sample	Crystallite size (nm)
1	MC_R0	14
	MC_R0.31	14
	MC_R2	13
	MC_R5	9
	MC_R10	9
2	MOP_R0	67
	MOP_R0.31	44
	MOP_R2	30
	MOP_R5	11
	MOP_10	12

**Figure 1.**
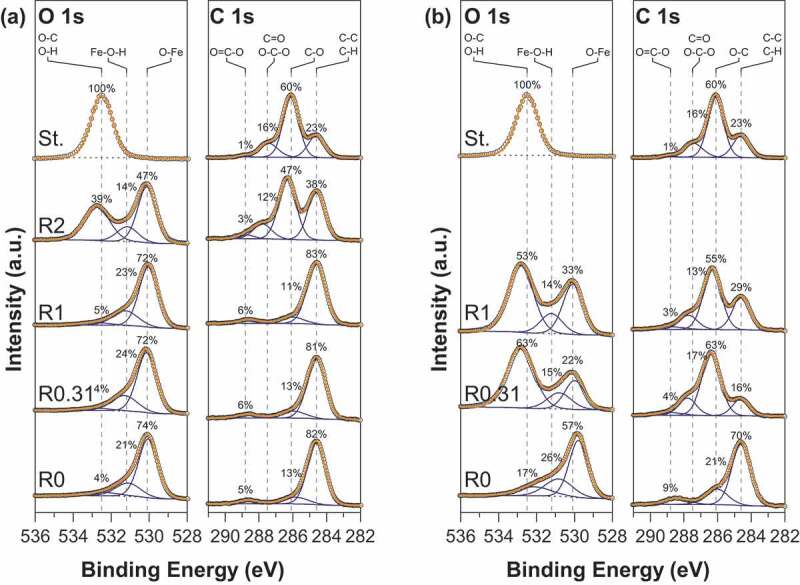
XPS high-resolution spectra of O 1s and C 1s core level for MC (a) and MOP (b) samples prepared at different R = m_starch_/m_Fe_ ratios. In each figure the O 1s and C 1s spectra of starch (St.) are also displayed.

The modification of the surface properties of magnetite nanoparticles upon starch functionalization was further examined by XPS. The C 1s and O 1s core-level spectra of pristine magnetite, starch-functionalized magnetite and gelatinized starch are shown in Figure 1. The O 1s spectra of pristine magnetite (R0) using routes 1 and 2 can be fitted with three components ascribed to Fe-O within Fe_3_O_4_ at ~530.0 eV, OH groups within Fe-OH at ~531.1 and C-O and/or water molecules on the surface at ~532.5 eV [22]. The detailed fitting parameters are reported in supporting information. Starch- functionalized magnetite nanoparticles show a significant contribution in their O 1s spectra at around 532.8 eV that characterizes O-C bonding in starch. The intensity of the O-C contribution clearly depends both on the synthesis route and the R ratio. The C 1s spectra of pristine magnetite nanoparticles (R0) display a major contribution at around 284.6 eV, and some small features near 286.2 eV and 288.6 eV corresponding to C-C/C-H; C-O and C=O, environments, respectively. The presence of carbon at the pristine magnetite nanoparticles surface reflects some contamination that is always observed at the surface of air-exposed samples. Functionalized nanoparticles exhibit a strong increase of the contributions at 286.2 eV and 288.8 eV characteristic of C-O/OH and O-C-O functional groups in starch, respectively. Here again, the starch contributions depend on the synthesis route and the R value. Interestingly, the presence of starch at the surface magnetite particles is observed at a much higher R ratio for the co-precipitation method (route 1) than for the oxidation method (route 2). In fact, from Figure 1 it can be concluded that surface properties of starch-functionalized magnetite nanoparticles are close to those of starch for R ≥ 0.31 and R ≥ 2 using routes 1 and 2, respectively.

**Figure 2.**
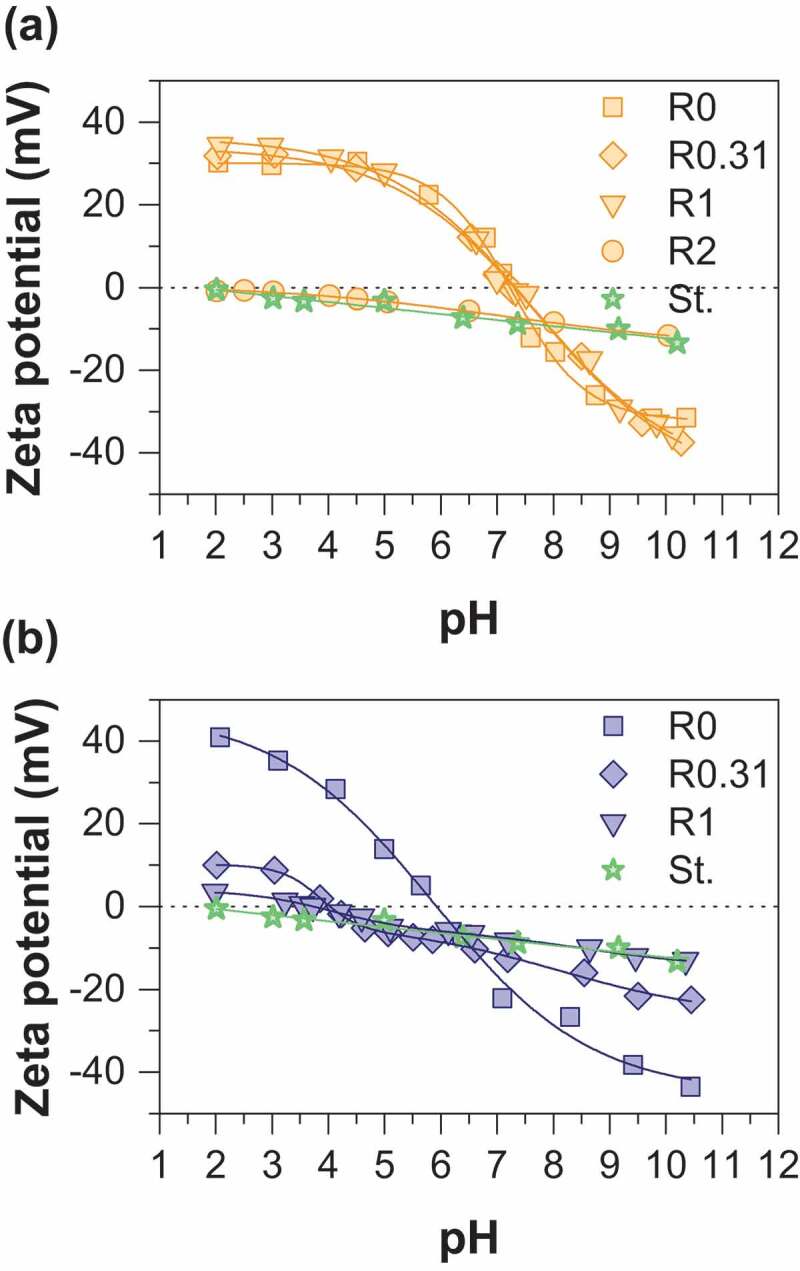
Zeta potential as a function of pH for (a) MC (b) MOP nanoparticles synthesized with different R = m_starch_/m_Fe_ ratios. In each figure the zeta potential curve of gelatinized starch (St.) is also displayed for comparison.

The zeta potential is an important parameter that allows to determine the stability of a colloidal suspension and the magnitude of the electrostatic interaction forces between particles. Figure 2 shows zeta potential vs pH curves of pristine magnetite, functionalized magnetite particles, and gelatinized starch. Gelatinized starch displays a weak negative surface charge due to some dissociation of protons from hydroxyl (OH) present in native starch and dissociation of carboxylic COOH groups introduced during gelatinization [23]. Irrespective of the synthesis route, the zeta potential of pristine magnetite evolves from negative values in the range [−40; −30 mV] at pH = 2 to positive ones in the range [30; 40 mV] at pH = 10.5. However, the resulting isoelectric point (IEP) differs significantly: values of 7.2 and 6.0 are obtained for routes 1 and 2, respectively. Kosmulski [24] compiled literature data and gave evidence for the influence of magnetite synthesis conditions on the IEP, values generally being in the range of 6.5–7.7 and 6.3–6.9 for routes 1 and 2, respectively. The precise measurement of isoelectric point of magnetite is generally challenging because this mineral is unstable in contact with atmospheric oxygen and the extreme surface of this mineral can be converted into maghemite (γ-Fe_2_O_3_). IEP values in the range of 5.5–7.5 have been usually reported for maghemite [24]. The difference in the IEP obtained may thus be ascribed to some uncontrolled surface oxidation of magnetite into maghemite during the synthesis even if great care was taken to limit the oxidation. The zeta potential and IEP values of starch-functionalized nanoparticles greatly differ from those of pristine nanoparticles, the effect of surface functionalization being more pronounced for magnetite prepared by route 2. In fact, due to starch coating, the zeta potential strongly decreases over the whole pH range with increasing R values and a weakly charged surface is obtained for sample MOP_R1. In addition, the IEP is drastically shifted to lower values of around 4.2 and 3.8 for MOP_R0.31 and MOP_R1, respectively. The zeta curves of samples prepared by route 1 display a different behavior: the zeta potential curves remain very similar to those of pristine magnetite for samples corresponding to R = 0.31 and R = 1. In contrast, the curve for MC_R2 closely matches that of pure gelatinized starch. Finally, the obtained results are in agreement with XPS analysis, the decrease in the zeta potential is perfectly correlated to the appearance of the characteristic chemical C-O/OH and O-C-O bonding of starch at the sample surface. A marked difference between the two synthesis procedures is the pH condition. In particular, modification of starch behavior due to partial hydrolysis under acidic conditions may occur (route 1) and this point will require further attention in future work. In any way, depending on the R values, the observed behaviors strongly suggest a specific interaction between the surface of magnetite and starch molecules. Evidently, it would be expected that the modification of the surface of magnetite by starch will affect the reactivity of the nanoparticles towards arsenic species.

### 3.2. Effect of sorbent dose

**Figure 3.**
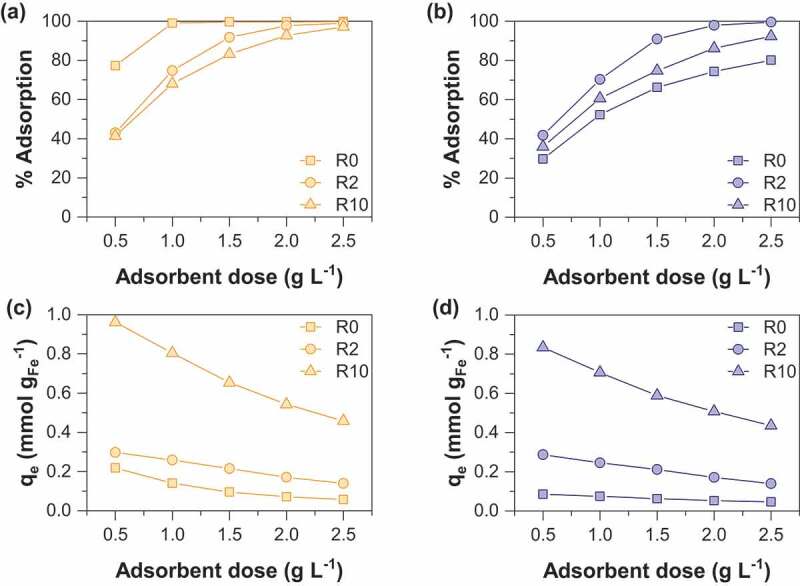
Comparison of As(V) adsorption percentage as a function of sorbent dose for (a) MC and (b) MOP samples synthesized with different R = m_starch_/m_Fe_ ratios and corresponding As(V) sorption capacities for (c) MC and (d) MOP samples. Experimental conditions: 0.1 M KCl; pH = 6; initial concentration of As(V) is 0.1 mM.

Arsenate removal percentage and sorption capacities as a function of sorbent dose are displayed in Figure 3. It is not surprising that the arsenate removal percentage increases with sorbent dose whatever the synthesis route used. This observation may be easily interpreted as the result of an increase in the number of active sorption sites with increasing the mass of adsorbent. Note that the adsorption of arsenate by starch is negligible (data not shown). MOP samples display increasing As(V) removal percentage in the following order R0< R10< R2 while the order is R10< R2< R0 for MC samples. Both nanoparticle size and the amount of magnetite in the adsorbent should be considered together to elucidate the sorption behavior. In fact, a decrease in the particle size is clearly observed with increasing R values (Table 1); the specific surface area and thus the number of active sorption sites increase with R. However, it is important to note that the proportion of magnetite in the adsorbent decreases with increasing R values. Thus, the balance between these two opposite effects induces the observed maximum arsenate removal percentage at the R value of 2 for MOP samples. This result agrees well with the previous study of An et al (2011) [13] on As(V) removal by starch magnetite nanoparticles prepared by coprecipitation of Fe(II) and Fe(III). The magnetite nanoparticles obtained from route 1 display a different behavior. It can thus be concluded that the decrease in the magnetite proportion in the adsorbent at increasing R values is the major factor that controls the adsorption removal percentage (R0> R2> R10). Figure 3(c) and 3(d) show the As(V) sorption capacity per unit mass of iron as a function of the adsorbent dose to eliminate the influence of the magnetite proportion in the adsorbent. It clearly appears that the sorption capacity increases with the R value. Therefore, enhanced adsorption of arsenic onto starch-functionalized magnetite nanoparticles is due to the decrease of particle size effect induced by starch.

### 3.3. Sorption kinetics

**Figure 4.**
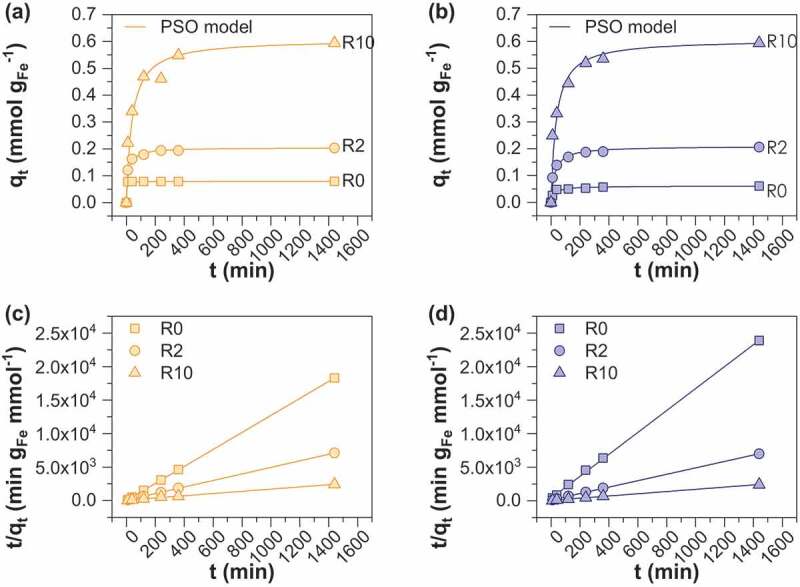
Effect of contact time and R ratio on adsorption of As(V) on (a) MC and (b) MOP samples. The curves (c) and (d) displayed pseudo-second-order linear kinetic modeling of As(V) on MC and MOP samples respectively. Experimental conditions: 0.1 M KCl; pH = 6; initial concentration of As(V) is 0.1 mM.; adsorbent dose = 1.5 g L^−1^.

The kinetics of As(V) sorption onto starch-functionalized MOP and MC magnetite was determined by varying the contact time from 0 to 1400 min (Figure 4) using 1.5 g L^−1^ adsorbent at an As(V) concentration of 0.1 mmol L^−1^. All samples have a similar sorption pattern except MC_R0 for which complete removal of As(V) occurred within the first 30 min thus leading to a constant sorption capacity of 0.08 mmol g_Fe_^−1^. All other samples display a rapid initial removal rate and follow then a rather slower adsorption uptake until an equilibrium is reached. Table 2 shows the time required to achieve 50% (t_50_) and 95% (t_95_) of arsenic removal. Fifty percent of As(V) removal efficiency is achieved within 40 minutes for all samples while 7, 8 and 11 h are required to achieve 95% of removal efficiency for MOP_R0, R2 and R10 and 22 min, 4 and 12 h for MC_R0, R2 and R10, respectively. For subsequent experiments and for the sake of convenience, an equilibration time of 24 h was chosen. Such a kinetic behavior is expensively reported in the literature [25–27] and is generally explained by the presence of a large number of sorption sites at the beginning of the sorption experiments followed by the gradual decrease in the sorption sites availability due to the increase in the repulsive forces between the already sorbed species and those in solution [26]. In addition, the adsorption capacities at equilibrium clearly increase with R, *i.e*. from 0.06 (MOP_R0) and 0.08 (MC_R0) to 0.60 mmol g_Fe_^−1^ for MOP_ and MC_R10 samples. The increase in sorption capacities with R support the assumption that starch acts by decreasing the particle sizes of adsorbents and that they mainly behave as non-agglomerated particles. The commonly used first and pseudo-second-order models were evaluated to simulate the kinetic data. The Pseudo-Second Order (PSO) kinetic model provided excellent fitting of all of the data (Table 3). The obtained PSO rate constants are much higher for samples synthesized using route 2 and decrease with increasing R ratio. This behavior may be interpreted as a consequence of lower diffusion rate of arsenite species into the layer of starch with increasing R ratio and thus reflecting more difficulties to access the active sites on magnetite surface.

**Table 2.** Values of t_50_ and t_95_ corresponding to the times at which the adsorption capacity is 50% and 95% of the steady-state values, respectively.

		*t_50_*	*t_95_*
Sample		(min)	(min)
MOP	R0	23	442
	R2	24	470
	R10	35	668
MC	R0	<10	22
	R2	13	256
	R10	38	734

**Table 3.** Pseudo first order (PFO) and pseudo-second-order (PSO) models for As(V) sorption onto MC and MOP samples; slopes and intercepts of the graphs for PSO model are presented in Figure 4c and d and were used to determine reaction rate constant k_2_ (g mmol^−1^ min^−1^) and amount of arsenic adsorbed at equilibrium q_e,cal_ (mmol g_Fe_^−1^); R^2^ is the correlation coefficient.

		MC			MOP		
Governing equation	Parameter	R0	R2	R10	R0	R2	R10
PFO *q_t_ = q_e_ (1-e-^k^_1_^t^)*	*q_e_* (mmol/g _Fe_^−1^)	0.003	0.09	0.40	0.03	0.12	0.39
	*k_1_* (min^−1^)	0.007	0.008	0.006	0.006	0.006	0.006
	*R^2^*	0.232	0.807	0.877	0.811	0.843	0.907
PSO	*q_e_* (mmol/g _Fe_^−1^)	0.08	0.20	0.61	0.06	0.21	0.60
	*k_1_* (min^−1^)	11.00	0.36	0.04	0.70	0.19	0.05
	*R^2^*	1.000	0.999	0.998	1.000	1.000	0.999

### 3.4. Adsorption isotherms

**Figure 5.**
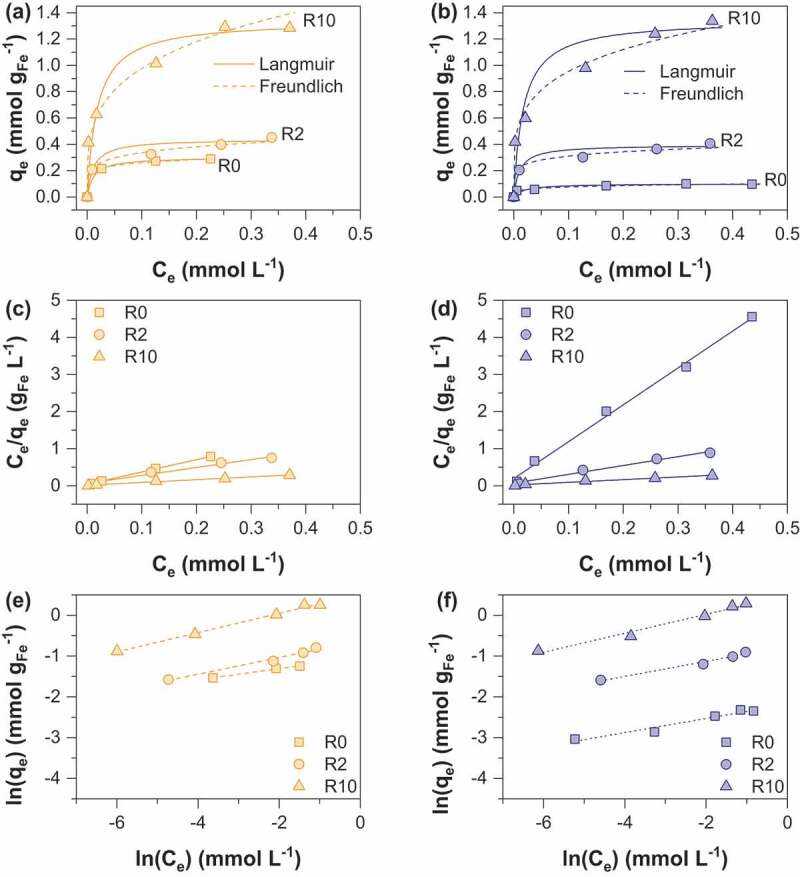
Adsorption isotherms of As(V) onto (a) MC (b) MOP samples synthesized at R = 0, 2 and 10; representation of the corresponding linear Langmuir relationship onto MC (c) and MOP (d) samples; representation of the corresponding linear Freundlich relationship onto MC (e) and MOP (f). Experimental conditions: 0.1 M KCl; pH = 6; adsorbent dose = 1.5 g L^−1^.

**Figure 6.**
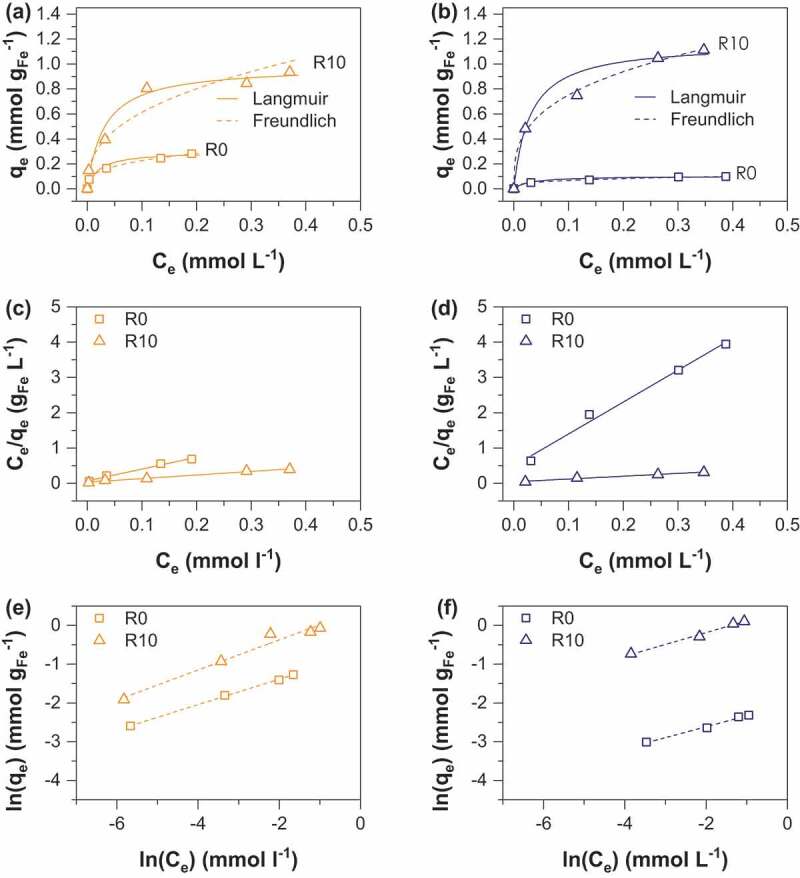
Adsorption isotherms of As(III) onto (a) MC (b) MOP samples synthesized at R = 0 and 10; representation of the corresponding linear Langmuir relationship onto MC (c) and MOP (d) samples; representation of the corresponding linear Freundlich relationship onto MC (e) and MOP (f). Experimental conditions: 0.1 M KCl; pH = 6; adsorbent dose = 1.5 g L^−1^.

As(V) and As(III) sorption isotherms were obtained at pH 6 and 1.5 g. L^−1^ sorbent dose and are displayed in Figures 5 and 6 respectively. As expected, isotherms show a gradual increase of sorption capacity with increase equilibrium concentration [13,25,28]. The classical Langmuir [29] (Equation (2)) and Freundlich [30] (Equation (3)) models were used to fit the experimental data.

(2)
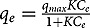

(3)
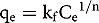

where q_e_ is the amount of As(V) adsorbed per unit mass of iron (mmol g_Fe_^−1^) at the equilibrium concentration C_e_ (mmol L^−1^), q_max_ corresponds to the maximum sorption capacity, k_F_ is the Langmuir constant related to the adsorption energy. The Freundlich constant k_F_ is the sorption capacity of the sorbent and n is the sorption intensity representing the degree of dependence of adsorption with equilibrium concentrations. Calculated parameters using linear regression analysis are shown in Tables 4 and 5 for As(V) and As(III) respectively. High correlation coefficients indicate that As(V) and As(III) sorption can be well described by the Langmuir and the Freundlich isotherms. A number of previous studies using iron oxides as an adsorbent have accordingly reported that arsenic adsorption fits both Langmuir and Freundlich models, in particular magnetite [31,32] and amorphous iron oxide [33,34]. The Langmuir model was used to determine the adsorption maxima q_max_ (mmol g_Fe_^−1^). The obtained q_max_ values for As(V) increase from 0.10 to 1.34 mmol g_Fe_^−1^ for MOP_R0 to R10 and 0.29 to 1.31 mmol g_Fe_^−1^ for MC_R0 to R10 (Table 4). In addition, q_max_ values for As(III) are 0.11 and 1.16 for MOP_R0 and R10 while 0.28 and 0.98 mmol g_Fe_^−1^ values are obtained for MC_R0 and R10, respectively (Table 5). Interestingly, pristine magnetite nanoparticles obtained from route 1 exhibits a ~3 times higher As(V) and As(III) maximum sorption capacity than pristine magnetite nanoparticles prepared from route 2. Assuming spherical nanoparticles with a diameter equal to the crystallite size determined by XRD (MC_R0 d_p_:14 nm; MOP_R0 d_p_:67 nm) one would expect that the specific surface area, and thus the adsorption capacity of MC_R0 would be ~4.8 times higher than for MOP_R0. Then, particles agglomeration in MC_R0 sample has to be invoked to explain lower q_max_ ratio. To our knowledge, the q_max_ values for pristine magnetite nanoparticles agree well with those reported in the literature. In particular, Yean et al. [35] obtained As (V) and As(III) q_max_ values of 0.101 and 0.389 mmol g^−1^ for commercial magnetite of a mean size of 20 nm. Ohe et al. [36] reported sorption capacities of 0.228 mmol g^−1^ for As(V) and 0.277 mmol g^−1^ for As(III) for magnetite obtained by the coprecipitation method. Starch-functionalized magnetite nanoparticles exhibit a much greater sorption capacity than pristine magnetite nanoparticles and the sorption capacity increases with R. Similar As(V) q_max_ values are obtained for the two routes at R = 2 and 10, whereas the As(III) q_max_ value at R = 10 is slightly lower for MC samples (Table 1). The decrease in particles size and the limitation of nanoparticles aggregation probably explain the increase in q_max_ values by a factor higher than 13 observed for route 2 while d_p_(MOP_R0)/d_p_(MOP_R10) equal only ~5.8. In contrast to route 2, the variation in size particles due to starch functionalization is limited for route 1 (d_p_(MC_R0)/d_p_(MC_R10) ~ 1.6) and thus the limitation of particles aggregation mainly explains the increase in q_max_ by a factor 4.5. The maximum As(V) and As(III) sorption capacities obtained in the present study are therefore close to 1.4 mmol g_Fe_^−1^at pH 6.0 for coprecipitated magnetite of 9 nm particles size stabilized with starch. The comparison of our results with literature data is difficult in view of the different experimental conditions and different properties of the adsorbent (particle size and stabilizer/adsorbent ratio) which strongly influence the obtained q values. However, Yean et al. (2005) reported a q value of 2.3 mmol/g using magnetite of 11 nm stabilized with oleic acid at pH 6. An et al. (2011) [13] obtained a q value of 3.3 mmol g^−1^ using starch-functionalized magnetite at pH = 5. Bisht and Neupane [37] achieved a q value of 1.65 for As(III) removal by starch magnetic nanoparticles of 16 nm particles size at pH = 6. The obtained results in the present study are particularly important because they demonstrate the adsorbent efficiency towards both As(III) and As(V); the obtained results for As(III) trapping are particularly interesting as it is well known to be more toxic and more difficult to remove from water than As(V). The maximum sorption capacities (mg/g iron oxide) of MOP and MC samples in comparison to those previously published in the literature are summarized in Table 6.

**Table 4.** Langmuir and Freundlich isotherm parameters for As(V) sorption on MC and MOP samples.

		MC			MOP		
Model	Parameters	R0	R2	R10	R0	R2	R10
Langmuir	*q_max_*	0.29	0.44	1.31	0.10	0.39	1.34
	*k_L_*	390	87	77	77	94	57
	*R^2^*	0.997	0.979	0.990	0.994	0.983	0.980
Freundlich	*k_F_*	0.35	0.51	1.82	0.11	0.43	1.62
	*n_F_*	7.7	5.5	3.7	5.4	6.9	4.3
	*R^2^*	0.999	0.983	0.988	0.987	0.967	0.927

q_max_ (mmol g Fe^−1^); 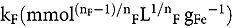.

**Table 5.** Langmuir and Freundlich isotherm parameters for As(III) sorption on MC and MOP samples.

		MC		MOP	
Model	Parameters	R0	R10	R0	R10
Langmuir	*q_max_*	0,28	0,98	0,10	1,16
	*k_L_*	82,9	32,2	33,2	33,7
	*R^2^*	0,976	0,988	0,979	0,971
Freundlich	*k_F_*	0,48	1,50	0,13	1,57
	*n_F_*	3,0	2,6	3,5	3,1
	*R^2^*	0,998	0,958	0,998	0,997

q_max_ (mmol g Fe^−1^); 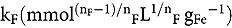.

**Table 6.** Comparison of As(V) and As(III) adsorption capacities.

Sorbent	pH	As(V) q_max *_	As(III) q_max *_	Ref.
Magnetite NPs	6	7.6	29.1	[32]
Magnetite NPs	6.6	17.1	20.7	[33]
Hollow αα-Fe_2_O_3_	-	75.3	58.6	[49]
Ferrihydrite	4.6	555	1330	[50]
Fe_3_O_4_ with Chitosan (R ~ 0,1)	-	12.2	-	[51]
Fe_3_O_4_ with Starch (R ~ 0,1)	5	179	-	[10]
	7	143	-	[10]
MOP R0	6	5.4	6.0	This study
MC R0	6	16.2	16.2	This study
MOP R10	6	74.8	68.3	This study
MC R10	6	72.6	53.1	This study

* mg/g_iron oxide._

### 3.5. Effect of pH on the adsorption of As(V) and As(III)

**Figure 7.**
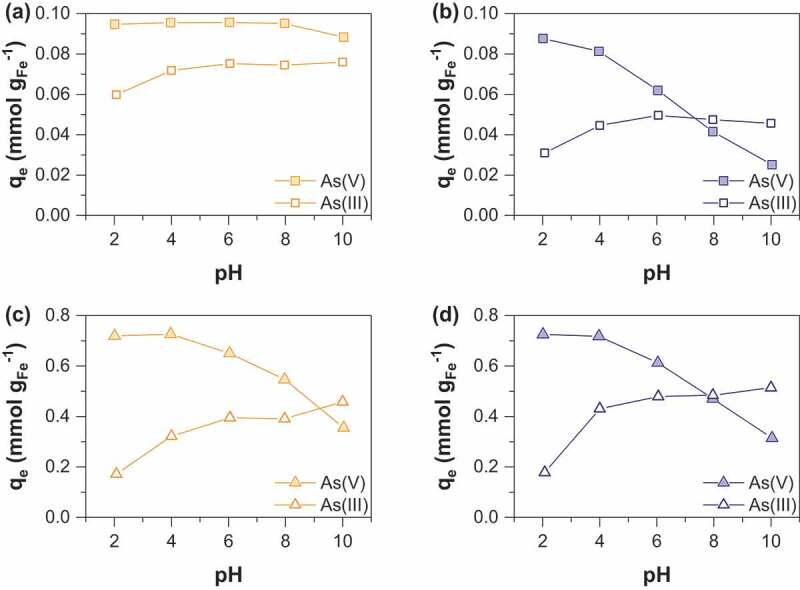
pH influence on adsorption of As(V) and As(III) by (a) MC (b) MOP samples synthesized at R = 0; pH influence on adsorption of As(V) and As(III) by (c) MC (d) MOP samples synthesized at R = 10. Experimental conditions: 0.1 M KCl; pH = 6; adsorbent dose = 1.5 g L^−1^; [As]_initial_ = 0,1 mM.

As(V) and As(III) sorption onto pristine and starch-functionalized magnetite nanoparticles as a function of pH are shown in Figure 7. First, it can be seen that the pristine magnetite samples resulting from route 1 (MC_R0) always display higher sorption capacities than the samples obtained from route 2 (MOP_R0) in the range of pH 2–10. Then, the sorption capacity of As(V) is strongly dependent on pH for MOP_R0 and decreases by increasing pH from 2 to 10. The As(V) sorption behavior as a function of pH agrees well with literature data on iron oxides [32,38,39] and zeta potential measurements (Figure 2). In our experimental pH range, the predominant species of As(V) are HAsO_4_^2-^ and H_2_AsO_4_^−^ (pK_a1_ = 2.2; pK_a2_ = 6.95; pK_a__3_ = 11.5). The removal of As(V) is favored in acidic pH conditions due to strong electrostatic attraction between negatively charged arsenate species and positive surface charge. In fact, the hydroxyl groups at the magnetite particles surface become more deprotonated when increasing pH which results in an increase of the net negative charge and disfavors arsenate adsorption. In addition, competition effect between hydroxide (OH^−^) and As(V) ions increases with pH. Electrostatic interactions seem thus to be a major factor for As(V) removal. However, As(V) sorption still occur at pH 10 thus indicating than other sorption processes, as surface complexation, may additionally occur. In contrast, only weak variations in sorption capacity of As(III) are observed in the pH range 4–10, whatever the synthesis route used. Again, pristine magnetite nanoparticles obtained from coprecipitation (MC_R0) display higher As(III) sorption capacity than magnetite prepared from the oxidation method (MOP_R0). As(III) species exist mainly as non-ionic or weakly charged forms in the pH range of 4–10, the first pKa value for aqueous As(III) species being 9.17 [40,41]. This implies that the repulsion forces between the magnetite particles and arsenic species are weak in most of the pH range studied and this suggests that sorption occurs through a complexation mechanism rather than electrostatic interactions. Many studies have demonstrated a different sorption behavior between As(III) and As(V) onto iron oxides and reported that As(III) sorption was not dependent on pH [38,42]. A strong decrease in As(III) sorption occurs at pH = 2. In this low pH range, other processes such as magnetite dissolution take place and may affect As(III) sorption by decreasing the number of active sites [40,41]. Starch-functionalized nanoparticles display the same behavior than pristine magnetite nanoparticles for both As(V) and As(III) sorption. However compared to pristine magnetite, the influence of the synthesis route onto the sorption capacity is much less pronounced. This may be explained by a much smaller difference in the crystallite size between the samples obtained from routes 1 (MC_R10) and 2 (MOP_R10) (Table 1). Finally, the crossover pH (*i.e*. the pH value at which As(V) and As(III) are equally sorbed) is about 7.5 for MOP_R0 and MOP_R10 samples (route 2), and about 9 for MC_R10 (route 1). As(V) sorption is thus more favorable than As(III) on a wide pH range and especially at low pH values. As discussed previously strong electrostatic repulsion between arsenate species and negatively charged surface sites at high pH values explain such a behavior. Similar results have been reported for arsenite and arsenate adsorption onto goethite and hydrous iron oxides (HFO) [42].

### 3.6. Effect of competing anions

**Figure 8.**
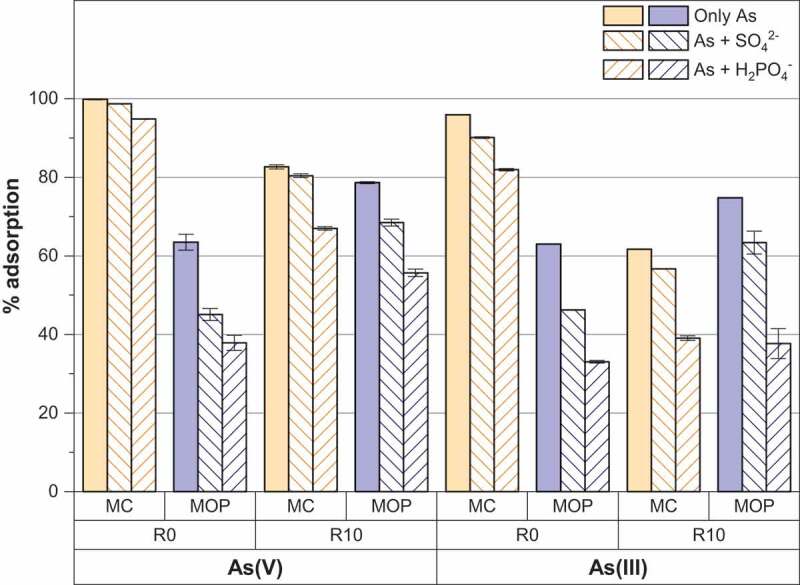
Effect of coexisting sulfate or phosphate anions on As(V) and As(III) adsorption percentage by MC and MOP samples synthesized with R = 0 and R = 10.

Phosphate and/or sulfate anions are commonly encountered in groundwater and are well known to sorb strongly on the surface of iron oxides [43,44]. So the presence of such anions can compete with arsenic anions for the adsorptive sites of magnetite nanoparticles. Figure 8 shows that As(V) and As(III) removal by magnetite nanoparticles (routes 1 and 2) is affected by the presence of sulfate and phosphate anions, a more pronounced effect always being observed for phosphate anions. The presence of 0.1 mM of sulfate or phosphate thus, respectively, decreases about 20% and 27% the As (V) removal onto pristine magnetite nanoparticles obtained from route 2 (MOP_R0). Decreases in the same order of magnitude are also observed for As(III) removal. Only a slight decrease of 5% is observed for As(V) removal by pristine magnetite nanoparticles obtained from route 1 (MC_R0) and the decrease did not exceed 15% for phosphate anions and As(III) removal. The same general conclusions are derived for starch-functionalized nanoparticles (routes 1 and 2). The observed interfering effect of sulfate and phosphate anions may thus be interpreted as a consequence of the chemical similarity of the anions studied. In particular, phosphate and arsenic are located in the same group of the periodic table, and their molecular structure is very similar. Roy et al. [27] studied the effect of phosphate on As(V) and As(III) on pristine magnetite particles and concluded that the effect of phosphate anions on As(V) was more significant to that of As(III). Here we observed a significant effect of sulfate and phosphate anions on both As(V) and As(III) removal. Other studies report a similar decrease in the adsorption of arsenate in the presence of phosphate onto hydrous ferric oxide, goethite and gibbsite [42,45,46].

### 3.7. Mössbauer analysis

**Figure 9.**
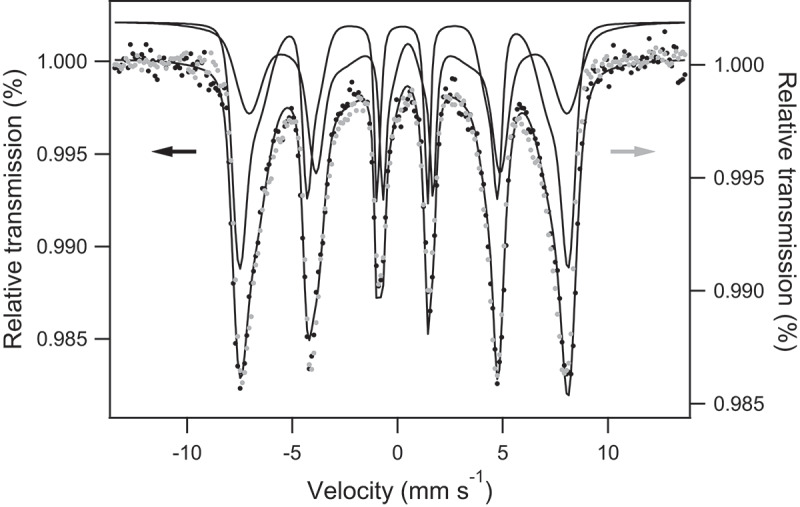
Room temperature Mössbauer spectrum of MC R0 magnetite before (black) and after (grey) As(V) adsorption.

Interactions leading to adsorption onto iron oxide surfaces can be hardly investigated by conventional spectroscopic techniques which are essentially bulk sensitive. However, Mössbauer spectra of phosphated maghemite [47] or phosphated magnetite [48] present contributions due to P-bonded iron when considering small particles (with an average grain size of 4.6 and 12 nm, respectively) for which the contribution of surface species is significant. Here, As–Fe interactions were tentatively explored considering As(V) adsorption onto MC_R0 magnetite whose average grain size is 14 nm (see Table 1). The broad sextet is fitted by hyperfine field distributions (HFDs) and the Voigt-based fitting (VBF) analysis was used to extract characteristic spectral parameters [15,45]. The MC R0 spectrum was fitted with two HFDs assigned to Fe ions located in tetrahedral and octahedral sites (center shift relative to α-iron = 0.27 mm s^−1^, respectively, 0.54 mm s^−1^, and average hyperfine field = 46.3 T, respectively, 41 T), see Figure 9. Sextet broadening is consistent with small magnetite nanoparticles where long-range magnetic order is perturbed.

After As adsorption ([As]_initial_ = 0.54 mM), Mössbauer spectrum recorded at 300 K and in transmission geometry is essentially unchanged (see Figure 9). Then, interestingly, contrary to their phosphated counterparts, our magnetite nanoparticles do not show any alteration of their Mössbauer fingerprint after As adsorption. Actually, it is well known that iron oxide affinity toward phosphate is strong. Mössbauer results reflect that iron oxide presents lower affinity to arsenate despite the fact that it is isoelectronic to phosphate: hyperfine interactions of the iron atoms at the surface of the nanoparticles remain unaltered.

## 4. Conclusions

In this study, we demonstrate great potential of starch-functionalized magnetite nanoparticles for both As(V) and As(III) removal. The primary findings and conclusions are summarized as follows:

(i) The starch coating renders the zeta potential close to neutrality over a wide range of pH and starch functionalization induces a decrease in crystallite size with increasing R = m_starch_/m_Fe_ ratio whatever the preparation method used.

(ii) Based on Langmuir isotherms, pristine magnetite nanoparticles (R = 0) that were synthesized by coprecipitation of a mixed Fe(II)/Fe(III) solution (MC samples) offer a ~ 3 times greater As(V) and As(III) sorption capacities than magnetite nanoparticles obtained by partial oxidation of an Fe(II) solution (MOP samples).

(iii) Starch-functionalized magnetite nanoparticles exhibit much greater As(V) and As(III) sorption capacities than pristine magnetite nanoparticles and sorption capacities increase with R values. At an R ratio of 10, the As(V) and As(III) sorption capacities determined from Langmuir isotherms are close to ~1.3 and ~1.0 for mmol g^−1^_Fe_ respectively whatever the synthesis method used which may be explained by the similar crystallite size for samples prepared at high R ratios.

(iv) The As(V) sorption capacity increases with decreasing pH due to increase in electrostatic attraction forces between positively charged surface sites and negatively charged arsenate species. As(III) sorption occurs through a complexation mechanism rather than by electrostatic interactions and is not pH dependent.

(v) The presence of anions such as sulfate or phosphate induces a decrease in both the As(V) and As(III) sorption capacities and this effect is much more pronounced for MOP samples.

(vi) Starch-functionalized magnetite nanoparticles display strong efficiency towards both As(V) and As(III) removal which may be particularly attractive as arsenic removal strategies often require As(III) oxidation into As(V) as a first step.

The obtained results thus encourage further investigation in the use of starch-magnetite nanoparticles in arsenic remediation strategies; the extra benefit of such a magnetic material is that it could be easily separated from contaminated water via a weak magnetic separator. Further work will therefore consider the recyclability and the reusability of starch-functionalized magnetite NPs for a cycle of adsorption process. A previous study [15] carried out with carboxymethyl cellulose (CMC) stabilized HFO, seems to suggest promising recycling properties of such stabilized iron oxide materials.
